# Coding variants in *RPL3L* and *MYZAP* increase risk of atrial fibrillation

**DOI:** 10.1038/s42003-018-0068-9

**Published:** 2018-06-12

**Authors:** Rosa B. Thorolfsdottir, Gardar Sveinbjornsson, Patrick Sulem, Jonas B. Nielsen, Stefan Jonsson, Gisli H. Halldorsson, Pall Melsted, Erna V. Ivarsdottir, Olafur B. Davidsson, Ragnar P. Kristjansson, Gudmar Thorleifsson, Anna Helgadottir, Solveig Gretarsdottir, Gudmundur Norddahl, Sridharan Rajamani, Bjarni Torfason, Atli S. Valgardsson, Jon T. Sverrisson, Vinicius Tragante, Oddgeir L. Holmen, Folkert W. Asselbergs, Dan M. Roden, Dawood Darbar, Terje R. Pedersen, Marc S. Sabatine, Cristen J. Willer, Maja-Lisa Løchen, Bjarni V. Halldorsson, Ingileif Jonsdottir, Kristian Hveem, David O. Arnar, Unnur Thorsteinsdottir, Daniel F. Gudbjartsson, Hilma Holm, Kari Stefansson

**Affiliations:** 1 deCODE genetics/Amgen, Inc., Reykjavik, Iceland; 2 Faculty of Medicine, University of Iceland, Reykjavik, Iceland; 3 Department of Internal Medicine, Division of Cardiovascular Medicine, University of Michigan, Ann Arbor, MI USA; 4 Department of Human Genetics, University of Michigan, Ann Arbor, MI USA; 5 School of Engineering and Natural Sciences, University of Iceland, Reykjavik, Iceland; 6 Department of Cardiothoracic Surgery, Landspitali University Hospital, Reykjavik, Iceland; 7 Department of Medicine, Akureyri Regional Hospital, Akureyri, Iceland; 8 Department of Cardiology, Division Heart & Lungs, University Medical Center Utrecht, University of Utrecht, Utrecht, The Netherlands; 9 HUNT Research Centre, Department of Public Health and General Practice, Norwegian University of Science and Technology, Levanger, Norway; 10 K.G. Jebsen Center for Genetic Epidemiology, Department of Public Health, Norwegian University of Science and Technology, Trondheim, Norway; 11 Department of Cardiology, St. Olav’s University Hospital, Trondheim, Norway; 12 Durrer Center for Cardiovascular Research, Netherlands Heart Institute, Utrecht, The Netherlands; 13 Institute of Cardiovascular Science, Faculty of Population Health Sciences, University College London, London, UK; 14 Farr Institute of Health Informatics Research and Institute of Health Informatics, University College London, London, UK; 15 Departments of Medicine, Pharmacology, and Biomedical Informatics, Vanderbilt University Medical Center, Nashville, TN USA; 16 Division of Cardiology, Department of Medicine, University of Illinois at Chicago, Chicago, IL USA; 17 Center For Preventive Medicine, Oslo University Hospital and Medical Faculty, University of Oslo, Oslo, Norway; 18 TIMI Study Group, Division of Cardiovascular Medicine, Brigham and Women’s Hospital and Harvard Medical School, Boston, MA USA; 19 Department of Computational Medicine and Bioinformatics, University of Michigan, Ann Arbor, MI USA; 20 Department of Community Medicine, UiT The Arctic University of Norway, Tromsø, Norway; 21 Reykjavik University, Reykjavik, Iceland; 22 Department of immunology, Landspitali University Hospital, Reykjavik, Iceland; 23 Department of Medicine, Levanger Hospital, Nord-Trøndelag Hospital Trust, Levanger, Norway; 24 Department of Medicine, Landspitali University Hospital, Reykjavik, Iceland

## Abstract

Most sequence variants identified hitherto in genome-wide association studies (GWAS) of atrial fibrillation are common, non-coding variants associated with risk through unknown mechanisms. We performed a meta-analysis of GWAS of atrial fibrillation among 29,502 cases and 767,760 controls from Iceland and the UK Biobank with follow-up in samples from Norway and the US, focusing on low-frequency coding and splice variants aiming to identify causal genes. We observe associations with one missense (OR = 1.20) and one splice-donor variant (OR = 1.50) in *RPL3L*, the first ribosomal gene implicated in atrial fibrillation to our knowledge. Analysis of 167 RNA samples from the right atrium reveals that the splice-donor variant in *RPL3L* results in exon skipping. We also observe an association with a missense variant in *MYZAP* (OR = 1.38), encoding a component of the intercalated discs of cardiomyocytes. Both discoveries emphasize the close relationship between the mechanical and electrical function of the heart.

## Abstract (web summary)

Rosa Thorolfsdottir et al. report a genome-wide association study of atrial fibrillation in 29,502 cases and 767,760 controls from Iceland and the UK Biobank. They identify a significant association with coding variants in *RPL3L*, the first ribosomal gene implicated in atrial fibrillation, and *MYZAP*, an intercalated disc gene.

## Introduction

Atrial fibrillation is the most common arrhythmia of clinical significance with an estimated number of 33.5 million individuals diagnosed with atrial fibrillation globally in the year 2010^1^. It is associated with increased mortality and morbidity, particularly stroke and heart failure, and is responsible for substantial health care costs^1^. Atrial fibrillation is a complex disease that is characterized by both mechanical and electrical abnormalities of the atria that may be detected prior to diagnosis of the arrhythmia itself. The role of atrial myopathy and fibrosis in the development of atrial fibrillation is increasingly recognized and it has been postulated that these processes may contribute to cardioembolic stroke in the absence of arrhythmia^2^. Thus, identification of the early stages of atrial myopathy may allow for therapy to prevent progression to atrial remodeling, atrial fibrillation, and stroke^3^.

Genome-wide association studies (GWAS), assessing primarily common sequence variants, have yielded over 30 genetic loci that associate with atrial fibrillation^4^. Most of the associated variants are non-coding and the causative genes remain unknown but the closest genes reveal a polygenic process, implicating transcription factors, cardiac ion channels, myocardial, and cytoskeletal proteins in the pathogenesis of atrial fibrillation. In the pre-GWAS era, linkage mapping and candidate gene sequencing linked a number of rare sequence variants to atrial fibrillation, mostly in single cases or familial atrial fibrillation, including variants in cardiac ion channel genes^4^. These variants explain a small proportion of atrial fibrillation cases, and for many, the genetic evidence is not robust.

In the past few years, through GWAS based on whole-genome sequencing, we have identified three low-frequency coding variants that associate with atrial fibrillation^5–8^. All three variants are in structural genes, the myosin sarcomere genes *MYH6*^5^ and *MYL4*^6,7^ and the cytoskeletal gene *PLEC*^8^. These findings support the notion of an important relationship between myocardial mechanical integrity and the development of arrhythmias.

Here, we continue our search for variants associated with atrial fibrillation to shed further light on the pathophysiology of this common arrhythmia. We performed an atrial fibrillation GWAS using data from Iceland and the UK Biobank, focusing on rare and low-frequency coding and splice variants, with follow-up of the most significant variants in samples from Norway and the US.

## Results

### Associations with coding variants in *RPL3L* and *MYZAP*

We performed a meta-analysis on atrial fibrillation including 14,710 cases and 373,897 controls from Iceland and 14,792 cases and 393,863 controls from the UK Biobank^9^, focusing on variants annotated as having moderate or high impact on protein function (including moderate: missense, in-frame indel, splice-region, and high impact: splice-acceptor, splice-donor, frameshift, stop-gained, and stop lost variants)^10^. To account for the expected impact, we applied the significance thresholds of *P* < 5.1 × 10^−8^ for moderate and *P* < 2.6 × 10^−7^ for high-impact variants^11^.

We found two novel genome-wide significant atrial fibrillation associations in the gene *RPL3L* on chromosome 16, with the missense variant p.Ala75Val (allele frequency 3.65% in Iceland, OR: 1.19, *P* = 3.4 × 10^−12^) and the splice-donor variant c.1167+1G>A (allele frequency 0.61% in Iceland, OR: 1.52, *P* = 8.2 × 10^−10^). The two *RPL3L* variants are not correlated (*D*’ = 1, *r*^2^ = 0.00024), and when conditioned on each other, both associations with atrial fibrillation remained (Supplementary Table 1). To assess the relationship between *RPL3L* and atrial fibrillation further, we tested all 15 low-frequency coding variants in the gene for association with atrial fibrillation after conditioning on p.Ala75Val and c.1167+1G>A (significance threshold = 0.05/15 = 0.0033, Supplementary Table 2). One variant associated with atrial fibrillation with a *P*-value below this threshold but the association was not genome-wide significant in the meta-analysis. The *RPL3L* gene encodes a ribosomal protein (ribosomal protein like 3L) that is primarily expressed in skeletal muscle and heart unlike most ribosomal proteins, that are ubiquitously expressed^12^.

We also observed a suggestive association with the missense variant p.Gln254Pro in the gene *MYZAP* on chromosome 15 (allele frequency 1.08% in Iceland, OR: 1.36, *P* = 7.8 × 10^−8^) (Table 1). No other coding variant in *MYZAP* associates independently with atrial fibrillation (Supplementary Table 3). *MYZAP* encodes myozap, myocardial zonula adherens protein, primarily expressed in the heart in man and its homolog in the mouse has been localized to the intercalated discs^13^.

**Table 1:** Meta-analysis results for atrial fibrillation variants

	*MYZAP* p.Gln254Pro missense			*RPL3L* p.Ala75Val missense			*RPL3L* c.1167+1G>A splice-donor		
Rs name	rs147301839			rs140185678			rs140192228		
Position (hg38)	chr15:57632516			chr16:1953015			chr16:1945498		
Data set (cases/controls)	Freq %	OR (95% CI)	*P*	Freq %	OR (95% CI)	*P*	Freq %	OR (95% CI)	*P*
Iceland (14,710/373,897)	1.08	1.38 (1.20–1.60)	9.0 × 10^−6^	3.65	1.18 (1.09–1.28)	6.4 × 10^−5^	0.61	1.37 (1.14–1.65)	8.7 × 10^−4^
UK Biobank (14,792/393,863)	0.36	1.32 (1.10–1.57)	0.0023	3.37	1.20 (1.13–1.27)	1.2 × 10^−8^	0.31	1.71 (1.40–2.07)	6.9 × 10^−8^
**Meta-analysis: Iceland and UK**		**1.36 (1.21**–**1.52)**	**7.8** × **10**^**−8**^		**1.19 (1.13**–**1.25)**	**3.4** × **10**^**−12**^		**1.52 (1.33**–**1.74)**	**8.2** × **10**^**−10**^
Fourier (1238/11,562)	0.21	1.61 (0.46–5.69)	0.46	4.88	0.93 (0.55–1.59)	0.79	0.12	1.59 (0.13–20.01)	0.72
Vanderbilt (759/759)	0.46	7.03 (1.29–38.23)	0.024	3.00	1.33 (0.88–2.02)	0.18	0.43	1.61 (0.46–5.69)	0.40
Tromsø (714/698)	0.49	1.14 (0.39–3.32)	0.81	3.90	1.29 (0.87–1.90)	0.20	0.073	1.60^a^ (0.53–4.88)	0.30
HUNT (6493/63,142)	0.64	1.47 (1.14–1.89)	0.0027	2.87	1.22 (1.07–1.40)	0.0038	0.15	1.19 (0.71–2.00)	0.51
**Replication only (Fourier, Vanterbilt, Tromsø, and HUNT)**		**1.50 (1.18**–**1.91)**	**8.1** × **10**^**−4**^		**1.22 (1.08**–**1.37)**	**0.0011**		**1.33 (0.88**–**2.00)**	**0.17**
**Combined**		**1.38 (1.25**–**1.53)**	**3.3** × **10**^**−10**^		**1.20 (1.14**–**1.25)**	**1.7** × **10**^**−14**^		**1.50 (1.32**–**1.70)**	**5.0** × **10**^**−10**^

*Freq* frequency of minor allele, *OR* odds ratio, *95% CI* 95% confidence interval, *P* *P* value from case–control association analysis. Results from more than one cohort are in bold

Here we show association results for atrial fibrillation for the discovery and follow-up data sets and the joint analysis of all data sets (combined). The *P* value for heterogeneity analysis was 0.52 for p.Gln254Pro in *MYZAP*, 0.92 for p.Ala75Val in *RPL3L* and 0.65 for c.1167+1G>A in *RPL3L*

^a^No control was a carrier of the variant resulting in 2×2 table entry with a zero count. Effect and *P*-value was calculated with the R-package “metafor” by adding a small constant of 1/2 to the cells of the 2×2 table

To further assess these associations, we tested the three sequence variants in four additional sample sets of 9204 cases and 76,161 controls combined, from the Nord-Trøndelag Health Study (HUNT), the Further Cardiovascular Outcomes Research with PCSK9 Inhibition in Subjects with Elevated Risk (FOURIER) trial, the Vanderbilt atrial fibrillation Registry, and the Tromsø Study. Joint analysis of all data sets yielded genome-wide significant association with atrial fibrillation of all three variants, *RPL3L* Ala75Val (OR: 1.20, *P* = 1.7 × 10^−14^), *RPL3L* c.1167+1G>A (OR: 1.50, *P* = 5.0 × 10^−10^), and *MYZAP* p.Gln254Pro (OR: 1.38, *P* = 3.3 × 10^−10^) (Table 1).

Three other moderate or high-impact coding variants in the genes *MYH6*, *PLEC*, and *MYL4* (recessive model), previously reported by us, were genome-wide significantly associated with atrial fibrillation in this data set^5–8^.

### The p.Ala75Val in *RPL3L* associates with electrocardiogram measures

**Fig. 1:**
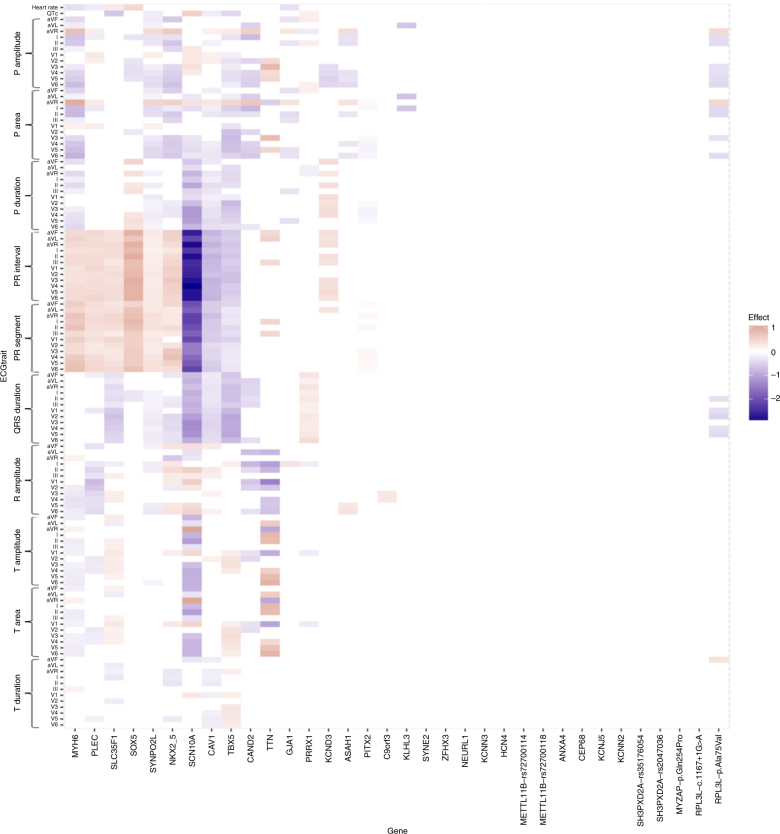
Heatmap showing the effects of atrial fibrillation variants on ECG traits in sinus rhythm ECGs, excluding atrial fibrillation cases. See Thorolfsdottir et al^8^. ECG measurements were available for 62,974 individuals without atrial fibrillation. Each column shows the estimated effect of the risk allele of an atrial fibrillation variant on various ECG traits. The effect of each variant, annotated with the corresponding gene name, is scaled with the log_10_-atrial fibrillation odds ratio. Red color represents a positive effect on the ECG variable and blue color a negative effect. The effect is shown only for significant associations after adjusting for multiple testing with a false discovery rate procedure for each variant. Non-significant associations are white in the heatmap

We have previously demonstrated that the effects of reported atrial fibrillation variants on ECG traits measured in sinus rhythm range from none to extensive and there is no clear relationship between effects on atrial fibrillation and effects on ECG measures^8^. For example, a sequence variant associated with atrial fibrillation in the sodium channel gene *SCN10A* has extensive and strong effects on ECG measures but a relatively small atrial fibrillation effect compared to the most significant common atrial fibrillation variant near *PITX2* that has minimal effect on ECG measurements (Fig. 1). Figure 1 shows the effects of the *RPL3L* and *MYZAP* variants on ECG traits compared to the effects of 31 published atrial fibrillation variants. For the analysis we used 289,297 sinus rhythm ECGs from 62,974 individuals not diagnosed with atrial fibrillation and tested all variants for association with 122 ECG variables, some of which are correlated (Supplementary Table 4 and Supplementary Data 1). We used the Benjamini–Hochberg false discovery rate (FDR) procedure controlling the FDR at 0.05 at each marker to account for multiple testing. The *RPL3L* missense variant p.Ala75Val associates with measures of atrial conduction during sinus rhythm, both P wave amplitude and area, and with QRS duration. Neither of the other variants in *RPL3L* and *MYZAP* associated with ECG traits in sinus rhythm. When testing for association with ECG traits using all ECGs irrespective of rhythm and history of atrial fibrillation, p.Ala75Val in *RPL3L* associates more significantly with ECG measurements and p.Gln254Pro in *MYZAP* associates with various P wave indices, R amplitude, and T wave indices (Supplementary Fig. 1).

### The p.Gln254Pro in *MYZAP* associates with sick sinus syndrome

Variants that associate with risk of atrial fibrillation also tend to associate with the related atrial arrhythmia sick sinus syndrome (SSS) and commonly with effects that are proportional to the atrial fibrillation effects^8^. One notable exception is the missense mutation in *MYH6* that we originally discovered through its association with high risk of SSS and confers a substantially greater risk of SSS than predicted from its effect on atrial fibrillation risk^5^. We tested the three new atrial fibrillation variants in 3568 SSS cases and 346,025 controls from Iceland and 403 cases and 403,181 controls from the UK Biobank^9^. In the joint analysis, p.Gln254Pro in *MYZAP* associates with SSS (OR: 1.65, 95% CI: 1.33–2.05, *P* = 5.0 × 10^−6^) (Supplementary Table 5).

To gain a better understanding of the new atrial fibrillation variants, we tested them for association with other phenotypes in deCODE´s genotype/phenotype database under both additive and recessive models but found no other associations passing Bonferroni correction. Association results for available relevant phenotypes including risk factors of atrial fibrillation are listed in Supplementary Tables 6 and 7. Since mutations in ribosomal genes are commonly associated with bone marrow failure, we specifically queried the relationship between the *RPL3L* variants and blood cells and found no associations. Similarly, since mutations in intercalated disc genes, albeit not *MYZAP*, have been associated with cardiomyopathies in man^14^ we assessed the link between the *MYZAP* variant and cardiomyopathies in our database, but found none.

### The splice-donor variant in *RPL3L* causes exon skipping

**Fig. 2:**
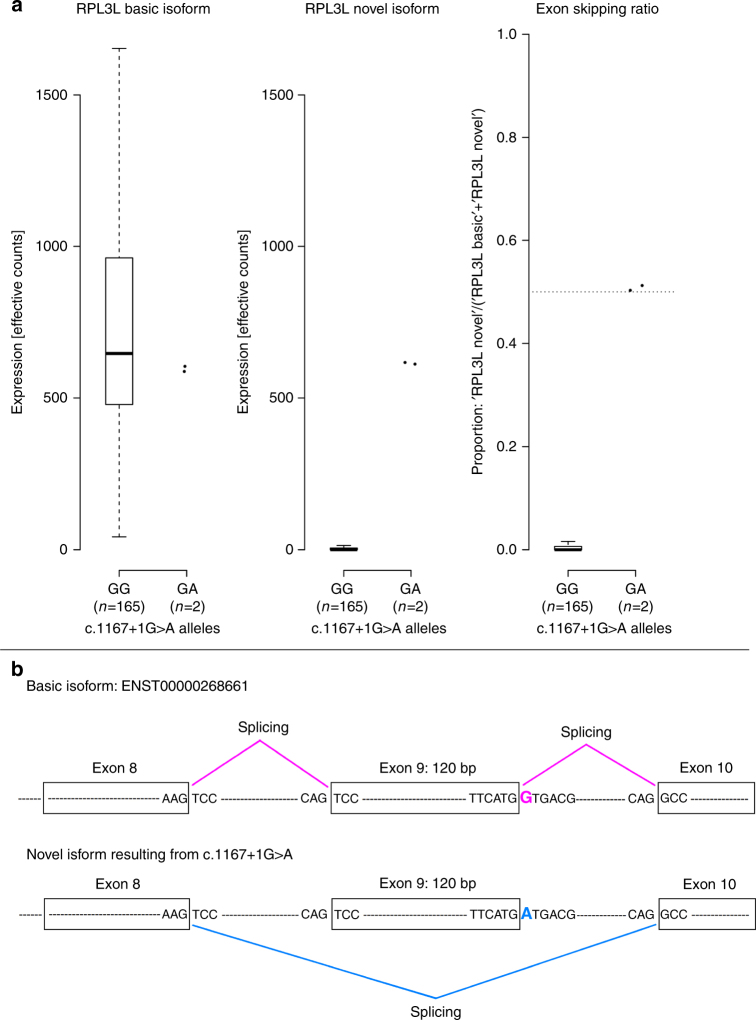
The effect of the splice-donor variant c.1167+1G>A in *RPL3L* on splicing. **a** Quantification of two forms of RPL3L transcripts; the primary isoform, ENST00000268661, and a novel isoform with skipping of exon 9 resulting from c.1167+1G>A. It also shows the proportion of novel isoform among all transcripts. A total of 167 samples, all from the right atrium, where included in the analysis. Two of those came from carriers of c.1167+1G>A. The figure demonstrates that only the two carriers have the novel isoform with skipping of exon 9. Their exon skipping proportion is ~0.5 while it is zero in non-carriers. **b** A schematic illustration of the splicing of RPL3L among carriers and non-carriers of c.1167+G>A. The variant is in a splice-donor site by the second last exon and results in exon skipping. The skipped exon is 120 base pairs and therefore its deletion is in-frame

We obtained RNA samples from cardiac atria of 167 Icelanders and used them to assess the effect of the splice-donor variant c.1167+1G>A in *RPL3L*. Two of the 167 individuals carry this variant. Non-carriers only produce the primary *RPL3L* isoform, but both carriers also produce an alternative isoform that skips exon 9 (*P* = 0.0052, Fig. 2a). We also found that carriers express the two isoforms in approximately equal abundance. Exon 9 is the second to last exon in *RPL3L* and is 120 base pairs long, and therefore its deletion is in-frame (Fig. 2b).

## Discussion

By performing a meta-analysis of atrial fibrillation using samples from deCODE and the UK biobank, focusing on rare and low-frequency coding and splice variants, with follow up in four sample sets from Norway and the US, we discovered three new atrial fibrillation variants in two genes, two in the ribosomal gene *RPL3L* and one in *MYZAP* that encodes a component of the cardiac intercalated discs. Risk of atrial fibrillation has not been associated with a ribosomal gene before.

The eukaryotic ribosome, composed of four different ribosomal RNAs and ~80 ribosomal proteins, is a complex cellular machine that translates messenger RNA into protein^15^. Only a few rare inherited diseases have been specifically linked to mutations in genes encoding ribosomal proteins. They include Diamond–Blackfan anemia and Shwachman–Diamond syndrome that are characterized by a distinct set of clinical features, including bone marrow failure and/or developmental abnormalities^16^. The ribosome has generally been considered to function in a housekeeping capacity but recent studies have revealed that ribosome activity may be regulated in a cell-specific manner, for example through changes in the protein composition of the ribosome^17,18^. One example is the *RPL3L* with expression restricted to skeletal muscle and the heart^12^. Ribosomes containing RPL3L instead of its ubiquitously expressed homolog, RPL3, have altered translational activity and it has been postulated that RPL3L may be a negative regulator of muscle growth^19^.

The *RPL3L* missense variant associating with atrial fibrillation is p.Ala75Val, and Ala75 is highly conserved in both *RPL3L* and *RPL3* over a range of species (PROVEAN impact prediction scores <−2.5^20^) (Supplementary Table 8). Sequencing of RNA samples from cardiac atria including from carriers of the splice-donor variant, c.1167+1G>A, demonstrated that the variant leads to skipping of *RPL3L* exon 9, the second to the last exon that encodes amino acid residues 350 to 389. These residues are 75% identical to the corresponding RPL3 residues. In yeast it has been shown that amino acids 373–380 in RPL3, corresponding to amino acids 382–389 in human RPL3L, form a part of the contact site of the ribosomes with the signal recognition particle that targets ribosomes to the endoplasmic reticulum membrane^21^. Based on functional similarities between RPL3 and RPL3L it is therefore possible that c.1167+1G>A disrupts engagement of RPL3L containing ribosomes with the endoplasmic reticulum and thus reducing ribosomal function. Since both *RPL3L* variants increase the risk of atrial fibrillation it could be predicted, based on the suggested effect of the splice-donor variant, that the variants are loss of, rather than gain of, function. The association of atrial fibrillation with a gene expressed in the atria that is involved with regulation of muscle growth is in line with the increasingly recognized tight link between mechanical myocardial integrity and the electrical function of the heart.

The *MYZAP* gene was recently discovered by Seeger et al. in an effort to find new components of the intercalated discs^13^, a highly specialized cell–cell contact structure that enables mechanical, electrical and chemical communication between cardiomyocytes. Human Myozap mRNA is primarily expressed in the heart and in the mouse the protein was predominantly found at intercalated discs and sarcomeric Z-discs^13^. In vitro functional studies revealed a role in cardiac signal transduction as Myozap promotes serum response factor signaling to the nucleus^13^. A knockdown of the Myozap ortholog in zebrafish and cardiac overexpression of Myozap in the mouse both resulted in cardiomyopathy^13,22^, suggesting an important role of the protein in maintaining cardiac integrity and function.

According to PROVEAN^20^, Gln254 is conserved and the variant is predicted to be deleterious (Supplementary Table 8). The variant is located at the edge of the Myozap protein region associated with both activation of serum response factor-dependent transcription and actin colocalization (amino acids 91–250), and could therefore potentially affect either one or both of these protein functions^13^. An introduction of proline, a conformationally constrained amino acid, can lead to perturbations in local folding and therefore might interrupt the function of adjacent domains.

Mutations in intercalated disc genes cause cardiomyopathies, in particular arrhythmogenic right ventricular cardiomyopathy, characterized by a notable risk of both atrial fibrillation and ventricular arrhythmias, and one of the leading causes of sudden cardiac death in young people and athletes^23^. Interestingly, conduction abnormalities and arrhythmias in arrhythmogenic right ventricular cardiomyopathy are commonly encountered before the appearance of structural defects^14^. Atrial fibrillation variants have also been identified in and close to genes encoding components of intercalated discs^4^, and the atrial fibrillation-associated gene *PITX2* has been shown to directly regulate intercalated disc genes^24^. P.Gln254Pro does not associate with cardiomyopathies, ventricular arrhythmias, or sudden cardiac death in our data, suggesting that it only affects the atria but we may lack power to identify a ventricular effect.

Like p.Gln254Pro in *MYZAP*, the three low-frequency missense and frameshift variants we have previously reported to increase the risk of atrial fibrillation, in *MYH6, MYL4*, and *PLEC*, also increase the risk of SSS^8^. Like *MYZAP*, all three genes encode structural components of the cardiomyocyte. In particular, *PLEC* encodes a multidomain cytoskeletal linking protein which, among other functions, connects with elements of the intercalated disc and has a role in maintaining its integrity^25,26^.

In summary, we report the association of three low-frequency coding variants in *RPL3L* and *MYZAP* with increased risk of atrial fibrillation. Using RNA samples from cardiac tissue we show that a splice-donor variant in *RPL3L* causes exon skipping. These results add to previous discoveries of three low-frequency coding variants in structural genes associating with atrial fibrillation and highlight the intricate connection between myocardial structure and arrhythmogenesis. The association of a missense variant in *MYZAP* with atrial fibrillation and SSS emphasizes the role of the intercalated discs in maintaining normal cardiac rhythm. The fact that a coding variant in a ribosomal protein specifically expressed in skeletal muscle and the heart increases risk of atrial fibrillation is in line with the novel concept of ribosome specialization in muscle and underscores the importance of this specialization for normal function of the heart. GWAS have linked a number of common variants with risk of atrial fibrillation but emerging discoveries of low-frequency coding variants associating with atrial fibrillation continue to shed new light on the pathogenesis of the disease.

## Methods

The study complies with the Declaration of Helsinki.

### Icelandic atrial fibrillation study population

The Icelandic atrial fibrillation sample consisted of 15,552 Icelanders diagnosed with atrial fibrillation (International Classification of Diseases (ICD) 10 code I.48 and ICD 9 code 427.3) according to electronic medical records at Landspitali, The National University Hospital, in Reykjavik, Iceland, and Akureyri Hospital, the two largest hospitals in Iceland, between 1987 and 2017. In total, 14,710 out of the 15,552 cases had genotypes and were included in the analysis. Controls were 373,897 Icelanders recruited through different genetic research projects at deCODE genetics. All participating subjects who donated blood signed informed consent. Personal identities of the participants and biological samples were encrypted by a third party system. The study was approved by the Icelandic Data Protection Authority and the National Bioethics Committee of Iceland (no. VSNb2015030021).

### UK Biobank atrial fibrillation study population

The UK Biobank project is a large prospective cohort study of ~500,000 individuals from across UK, aged between 40 and 69 at recruitment. The study has collected extensive phenotypic and genotypic information on its participants, including ICD coded diagnoses from inpatient and out-patient hospital episodes^9^. The atrial fibrillation population from UK Biobank consisted of 14,792 cases and 393,863 controls, all individuals of European ancestry recruited between 2006 and 2010^9^. Atrial fibrillation was ascertained based on ICD diagnoses. These are primary or secondary ICD-9 or ICD-10 diagnoses codes a participant has had recorded across all their episodes in hospital. Self-reported diagnoses were excluded from our analysis. Further details on the recruitment and variables collected in the UK Biobank study can be found in previous publications^9,27^.

### The Vanderbilt Atrial Fibrillation Registry

We genotyped novel atrial fibrillation variants in an atrial fibrillation sample set (759 cases and 759 controls) from the Vanderbilt Atrial Fibrillation Registry, a clinical and genetic registry at the Vanderbilt University Medical Center in Nashville, Tennessee. At enrollment into the registry, a detailed medical and drug history was obtained from all patients and patients were also asked to complete a symptom questionnaire. Patients with history of atrial fibrillation only associated with cardiac surgery were excluded from this study. Written informed consent was obtained from all patients under a protocol approved by the Vanderbilt University Institutional Review Board.

### FOURIER replication cohort

We also followed-up novel atrial fibrillation variants in an atrial fibrillation sample set originating from the Further Cardiovascular Outcomes Research with PCSK9 Inhibition in Subjects with Elevated Risk (FOURIER) trial (1238 atrial fibrillation cases and 11,562 controls). FOURIER is a randomized placebo-controlled, double-blind, parallel-group, multinational trial testing the hypothesis that adding the drug evolocumab to statin therapy will reduce the incidence of major adverse cardiovascular events in patients with clinically evident cardiovascular disease (CVD). The whole study group consisted of 27,564 patients recruited at 1242 cities in 49 countries from 2013 to 2015. Eligible patients were between 40 and 85 years of age and had clinically evident atherosclerotic CVD. The design of the trial has been described in detail elsewhere^28,29^. A subset of over 12,000 participants of European descent from the FOURIER trial have been genotyped by us by whole-exome sequencing, chip-typing, and imputation. Detailed phenotypic information was gathered on all FOURIER study participants, including atrial fibrillation disease status. The Fourier atrial fibrillation sample set consists of 1238 cases and 11,562 controls of European descent, excluding all Icelandic participants.

### Norwegian atrial fibrillation study population from the Tromsø Study

The Tromsø Study is a population-based prospective study with repeated health surveys in the municipality of Tromsø, Norway^30^. So far, more than 40,000 individuals have been examined.The population is being followed-up on an individual level with registration and validation of diseases and death and an endpoint registry has been established for CVD. Discharge diagnosis lists of CVD have been retrieved from the University Hospital of North Norway in Tromsø, and medical records for all individuals with a CV discharge diagnosis (including visits to out-patient clinics, out of hospital journals, autopsy records, and death certificates) have been reviewed. Atrial fibrillation has been registered based on ICD-9 and ICD-10 codes since 1986 as part of the ongoing CV endpoint registration in the Tromsø Study. People with postoperative atrial fibrillation only (≤28 days after the procedure) are registered, but are not included as cases. For the current project, one sex-matched and age-matched control for each case of atrial fibrillation from was drawn from the population-based Tromsø 4 survey. Participants in the Tromsø Study gave informed, written consent. The study was approved by the Regional Committee for Medical Research Ethics. The atrial fibrillation sample set consists of 714 cases and 698 controls.

### The Nord-Trøndelag Health Study

The Nord-Trøndelag Health Study (HUNT) is a population-based health survey conducted in the county of Nord-Trøndelag, Norway. Individuals were included at three different time points during ~20 years (HUNT1 (1984–1986), HUNT2 (1995–1997), and HUNT3 (2006–2008))^31^. At each time point, the entire adult population (≥20 years) was invited to participate by completing questionnaires, attending clinical examinations, and interviews. Participation rates have generally been high: 89.4% (*n* = 77,212), 69.5% (*n* = 65,237), and 54.1% (*n* = 50,807) in HUNT1, HUNT2, and HUNT3, respectively^31^. Taken together, the health studies included information from over 120,000 different individuals from Nord-Trøndelag. Biological samples including DNA have been collected for ~70,000 participants. Atrial fibrillation was defined based on ICD-10 codes collected from local hospitals and out-patient clinics between 1999 and 2016. Cases (6493) were defined as individual with one or more ICD-9 or ICD-10 codes specific for atrial fibrillation (“I48” or “427.3”) whereas controls (63,142) were all individuals without a code specific for atrial fibrillation.

### Secondary phenotypes

Novel atrial fibrillation variants were tested for association with other phenotypes in the deCODE genetics phenotype database which contains extensive medical information on various diseases and other traits. The pacemaker population sample set includes 3578 individuals who received a pacemaker implantation (NCSP surgical codes FPE and FPF) at LUH between 1997 and 2015. The SSS sample set includes 3578 individuals who received the diagnosis of SSS (ICD-10 code I49.5, ICD 9 code 427.8) at LUH in Reykjavik between 1987 and 2015. Ischemic stroke cases were identified from a registry of individuals diagnosed with ischemic stroke or transient ischemic attack (TIA) at LUH during the years 1993 to 2014 (*n* = 5626). The ischemic stroke or TIA diagnoses were based on standard WHO criteria and imaging evidence (either CT or MRI), and were clinically confirmed by neurologists. A total of 1369 individuals with ischemic stroke were classified as having cardioembolic stroke based on a neurologist review of medical records and classification according to the Trial of Org 10172 in Acute Stroke Treatment (TOAST)^32^. The controls used in the various case–control analyses of this study consisted of disease-free controls randomly drawn from the Icelandic genealogical database and individuals from other genetic studies at deCODE. We also assessed the association of novel atrial fibrillation variants with SSS among 403 cases and 403,181 controls in the UK Biobank.

### Electrocardiogram data

Electrocardiograms (ECGs) obtained in Landspitali, The National University Hospital, the largest and only tertiary care hospital in Iceland, have been digitally stored since 1998. We have analysed 434,000 ECGs from 88,217 individuals obtained between 1998 and 2015. To assess the effect of atrial fibrillation variants on ECG traits, and thus cardiac electrical function, in the absence of atrial fibrillation, we excluded ECGs from individuals with atrial fibrillation and pacemakers and used 289,297 sinus rhythm (heart rate 50–100 beats per min) ECGs of 62,974 individuals for the primary analysis. The ECGs were digitally recorded with the Philips PageWriter Trim III, PageWriter 200, Philips Page Writer 50, and Phillips Page Writer 70 cardiographs and stored in the Philips TraceMasterVue ECG Management System. These were ECGs obtained in all hospital departments, from both in patients and outpatients. Digitally measured ECG waveforms and parameters were extracted from the database for analysis. The Philips PageWriter Trim III QT interval measurement algorithm has been previously described and shown to fulfill industrial ECG measurement accuracy standards^33^. The Philips PR interval and QRS complex measurements have been shown to fulfill industrial accuracy standards^34^.

### Whole-genome sequencing, variant calling, and imputation in Iceland

In Iceland, the study is based on whole-genome sequence data from the whole blood of 15,220 Icelanders participating in various disease projects at deCODE genetics. In addition, 151,677 Icelanders have been genotyped using Illumina SNP chips and genotype probabilities for untyped relatives has been calculated based on Icelandic genealogy. The sequencing was done using Illumina standard TruSeq methodology to a mean depth of 35 (SD 8). Autosomal SNPs and INDEL’s were identified using the Genome Analysis Toolkit version 3.4.0^35^. Variants that did not pass quality control were excluded from the analysis according to GATK best practices^7^. Information about haplotype sharing was used to improve variant genotyping, taking advantage of the fact that all sequenced individuals had also been chip-typed and long-range phased^36^.

The informativeness of genotype imputation (imputation information) was estimated by the ratio of the variance of imputed expected allele counts and the variance of the actual allele counts:

$$\frac{{{\mathrm{Var}}({{E}}({\mathrm{\theta }}|{\mathrm{chip}}\;{\mathrm{data}}))}}{{{\mathrm{Var}}({\mathrm{\theta }})}}$$

where θ is the allele count. Here, Var(*E*(*θ*/chip data)) is estimated by the observed variance in the imputed expected counts and *Var*(*θ*) was estimated by *p*(1 − *p*), where *p* is the allele frequency.

Variants were annotated using Ensembl release 80 and Variant Effect Predictor (VEP) version 2.8^10^. A total of 32.5 million variants passed the quality threshold and were imputed into 151,677 Icelanders who had been genotyped using Illumina chips.

To account for inflation in test statistics due to cryptic relatedness and stratification, we applied the method of LD score regression^37^. With a set of 1.1M variants we regressed the *χ*^2^ statistics from our GWAS scan against LD score and used the intercept as a correction factor. The LD scores were downloaded from a LD score database (ftp://atguftp.mgh.harvard.edu/brendan/1k_eur_r2_hm3snps_se_weights.RDS; accessed 23.06.2015). The estimated correction factor for atrial fibrillation based on LD score regression was 1.39 for the additive model in the Icelandic sample and 1.04 in UK Biobank.

### Genotyping in the UK biobank data

In the UK Biobank, genotyping was performed using a custom-made Affymetrix chip, UK BiLEVE Axiom^38^, in the first 50,000 participants, and with Affymetrix UK Biobank Axiom array in the remaining participants^39^; 95% of the signals are on both chips. Imputation was performed by Wellcome Trust Centre for Human Genetics using a combination of 1000Genomes phase 3^40^, UK10K^41^, and Haplotype Reference Consortium (HRC) reference panels^42^, for up to 92,693,895 SNPs^43^.

### Single-variant genotyping

For genotyping of single variants in atrial fibrillation sample sets from the Vanderbilt registry, FOURIER trial, and Norway, we used the Centaurus (Nanogen) or KASP platforms.

### Statistical analysis

We performed a meta-analysis on 14,710 atrial fibrillation cases and 373,897 controls from Iceland and 14,792 atrial fibrillation cases and 393,868 controls from the UK Biobank. We used logistic regression to test for association between SNPs and atrial fibrillation and other phenotypes in the Icelandic study, treating phenotype status as the response and allele count as a covariate. We used allele counts from genotyping or integrated over possible genotype counts based on imputation. Other available individual characteristics that correlate with phenotype status were also included in the model as nuisance variables. In Iceland these covariates were: sex, county of birth, current age, or age at death (first and second order terms included), blood sample availability for the individual and an indicator function for the overlap of the lifetime of the individual with the time span of phenotype collection. In the UK biobank study 40 principal components were used to adjust for population stratification and age and sex were included as covariates in the logistic regression model. Only white British individuals were included in the study. For the meta-analysis we used a fixed-effects inverse variance method^44^ based on effect estimates and standard errors from the Icelandic and the UK Biobank study. Only sequence variants from the Haplotype Reference Consortium panel (HRC)^42^ were included in the meta-analysis and variants from deCODE and the UK Biobank imputation were matched on position and alleles. Standard errors were calculated in the following way:

For a *P*-value smaller than 1 we calculate the standard error as follows:

$$P = 2{\mathrm{\Phi }}\left( z \right) = 2{\mathrm{\Phi }}\left( {\frac{\beta }{\sigma }} \right).$$

Solving for *σ* gives

$$\sigma = \frac{\beta }{{{\mathrm{\Phi }}^{ - 1}\left( {\frac{P}{2}} \right)}}$$

If *P* = 1, then ${\mathrm{\Phi }}^{ - 1}\left( {\frac{P}{2}} \right) = 0$ and the above method breaks down. In this case we use data from other markers to estimate the relationship between allele frequency (*f*) and imputation information (*I*) and *σ* as follows:

$$Var\left( \beta \right) = \sigma ^2 \propto \frac{1}{N}f(1 - f) \propto \frac{1}{I}f(1 - f)$$

Sample size (*N*) is proportional to imputation information (*I*) if we are always basing the analysis on the same set of individuals. Therefore, if we fit the following linear model:

$$\log \left( {\sigma ^2} \right) = \gamma _1 + \gamma _I{\mathrm{log}}(I) + \gamma _f{\mathrm{log}}(f\left( {1 - f} \right))$$

for a subset of 100,000 markers spread over the genome with MAF ranging close to uniformly between 0.1 and 50% and info between 0.9 and 1 and pick the subset of markers with *P* < 0.9 then we can predict σ for a marker with *P* close to 1.

We corrected the threshold for genome-wide significance for multiple testing with a weighted Bonferroni adjustment using as weights the enrichment of variant classes with predicted functional impact among association signals estimated from the Icelandic data^11^.

With 32,463,443 sequence variants in the Icelandic data the weights given in Sveinbjornsson et. al. were rescaled to control the family-wise error rate. This yielded significance thresholds of 2.6 × 10^−7^ for high-impact variants (*N* = 8464) and 5.1 × 10^−8^ for moderate-impact variants (*N* = 149,983).

Conditional analysis of the region around novel atrial fibrillation variants, was performed by adding the top variant or variants as a covariate while testing every SNP in the region for association with atrial fibrillation in the Icelandic data.

We tested atrial fibrillation variants for association with 122 ECG measurements using linear regression, treating the ECG measurement as the response and the genotype as the covariate. Following the procedures described in Thorolfsdottir et al.^8^, ECG measurements were adjusted for sex, year of birth, and age at measurement and were subsequently standardized to have a normal distribution. For individuals with multiple ECG measurements, the mean standardized value was used. We assume that the quantitative measurements follow a normal distribution with a mean that depends linearly on the expected allele at the variant and a variance-covariance matrix proportional to the kinship matrix^45^. The Benjamini–Hochberg FDR procedure controlling the FDR at 0.05 at each marker was used to account for multiple testing.

### Expression analysis in cardiac tissue

RNA sequencing was performed on samples from cardiac right atrium of 167 Icelanders (see Supplementary Table 9, for subject characteristics). The samples were obtained during cardiothoracic surgery at Landspitali, The National University Hospital, in Reykjavik, Iceland. In the case of the splice-donor variant in *RPL3L* (c.1167+1G>A), the RNA samples from cardiac atria were used to identify a novel isoform and quantify expression at the transcript level. RNA sequencing libraries were inspected for sequencing and alignment integrity using parameters retrieved from RNA-SeQC^46^, Picard CollectRnaSeqMetrics (http://broadinstitute.github.io/picard/), and FastQC (http://www.bioinformatics.babraham.ac.uk/projects/fastqc). Genotype concordance was determined by comparing imputed genotypes to those derived from RNA-seq. Genome alignments were found using STAR^47^ aligning to GRCh38 with ensemble v87^48^ gene annotations. Alignments of RNA-seq reads of carriers of the variant contained several reads that spliced over exon 9 in transcript ENST00000268661 of *RPL3L*. Neither of the two other transcripts of *RPL3L* showed any expression in all samples (See Supplementary Fig. 2). To assess quantitatively the effect of the variant on the isoform usage we created the transcript sequence for the novel isoform, added it to the ensemble v87 transcriptome, and re-quantified all samples using kallisto^49^ and the modified transcriptome. The expression of the annotated and novel transcript was corrected w.r.t. the size factor computed from the gene expression analysis. Finally the proportion of novel isoform usage was computed by dividing the estimated expression of the novel isoform with the sum of the expression of both isoforms. Due to the small number of carriers, two samples out of 167, we opted for a conservative test for computing the significance of the observed event, that the carriers have a ratio of 50% vs near 0% for non-carriers. The test used was the two-sided Mann–Whitney *U* test, which only takes the relative ranks of the samples into account and not the underlying values. The *P*-value computed was *P* = 0.0052, the lowest possible *P*-value that can be obtained using this statistical test with *n*_1_ = 165 and *n*_2_ = 2.

### Data availability

The Icelandic population WGS data has been deposited at the European Variant Archive under accession code PRJEB8636. The authors declare that the data supporting the findings of this study are available within the article, its Supplementary Data files and upon request.

## Electronic supplementary material

Supplementary Information

Description of Additional Supplementary Files

Supplementary Data 1
