# Supplementary material for: Coding variants in RPL3L and MYZAP increase risk of atrial fibrillation
Source: Commun Biol. 2018 Jun 12;1:68. doi: 10.1038/s42003-018-0068-9 (PMC6123807; doi:10.1038/s42003-018-0068-9)
Supplement: Supplementary file 1 — Supplementary Information [file 42003_2018_68_MOESM1_ESM.pdf]

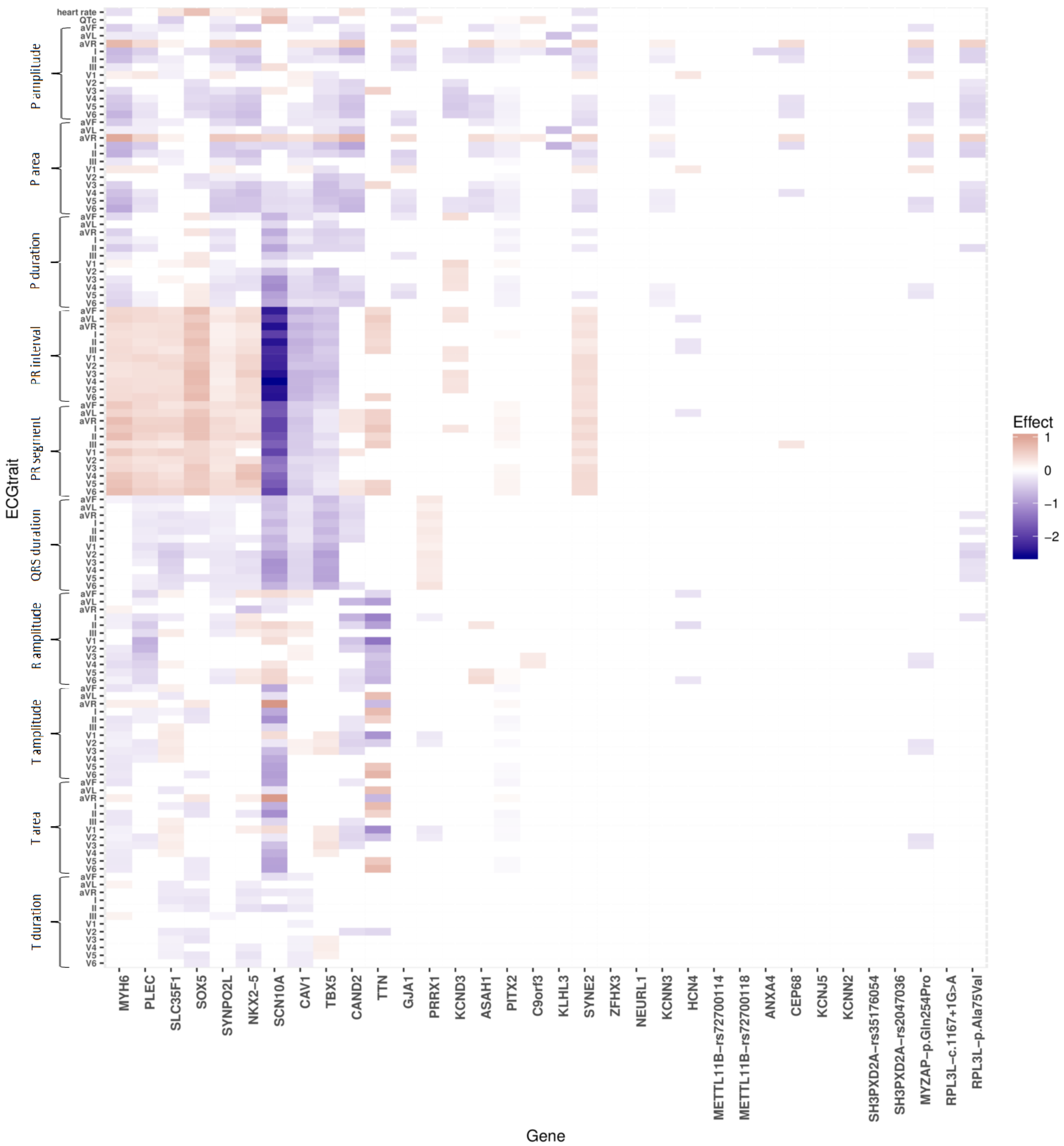

Supplementary Figure 1. Heatmap showing the effects of atrial fibrillation variants on electrocardiogram (ECG) traits of all ECGs, not excluding atrial fibrillation cases.[1] ECG measurements were available for 88,217 individuals. Each column shows the estimated effect of the risk allele of an atrial fibrillation variant on various ECG traits. The effect of each variant, annotated with the corresponding gene name, is scaled with the $\log_{10}$-atrial fibrillation odds ratio. Red color represents a positive effect on the ECG variable and blue colors a negative effect. The effect is shown only for significant associations after adjusting for multiple testing with a false discovery rate procedure for each variant. Non-significant associations are white in the heatmap.

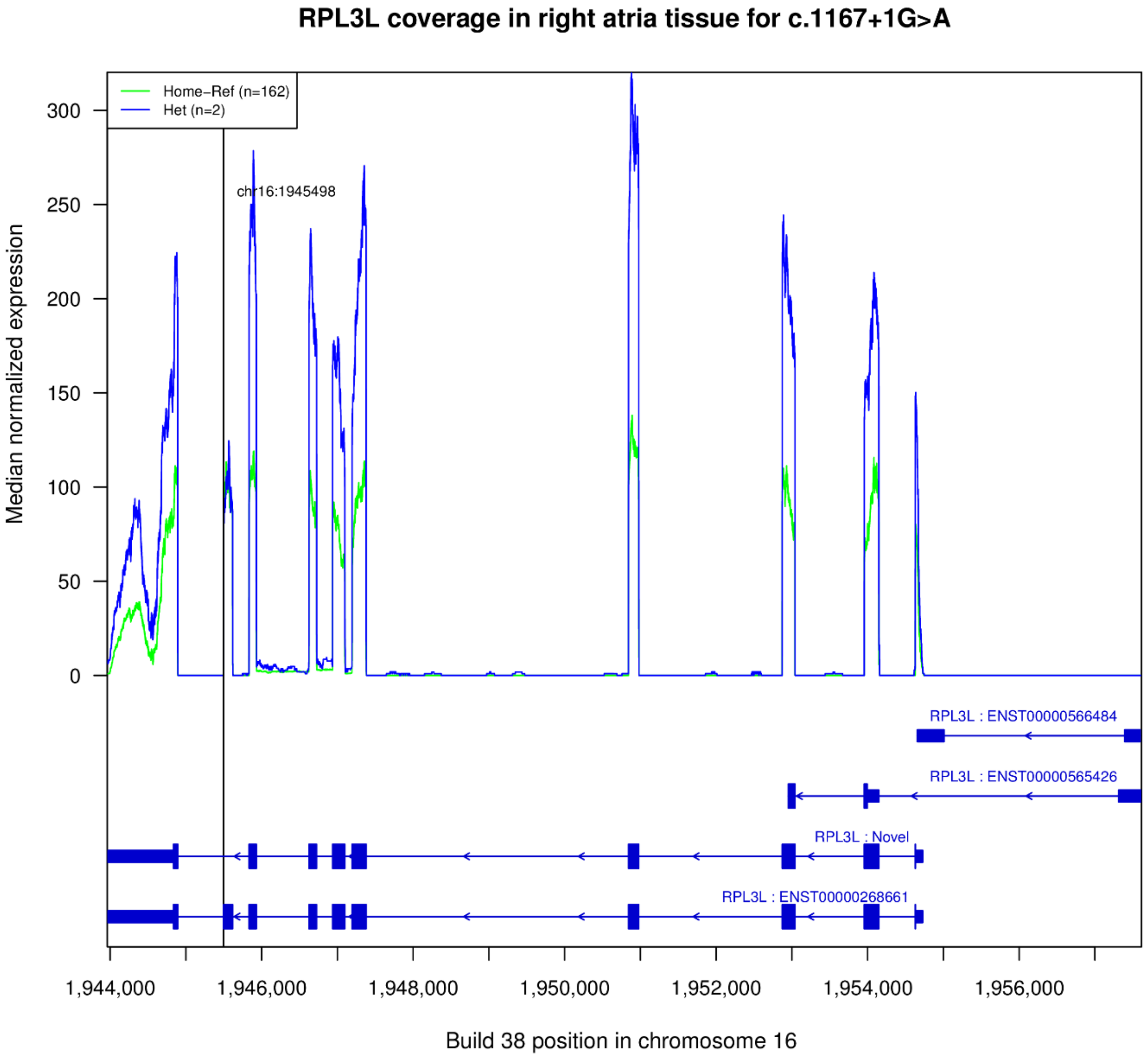

Supplementary Figure 2. Coverage of RNA reads aligning to the *RPL3L* locus, sequenced from right atrial tissue.

Position of the c.1167+1G>A mutation is labeled adjacent to exon 9 of ENST00000268661 transcript. No expression is detected for the two other annotated transcripts. Median coverage for c.1167+1G>A non-carriers (in green) is relatively even for all exons of ENST00000268661, while the expression of exon 9 is less for carriers (in blue) compared to other exons in the transcript.

Supplementary Table 1: Conditional analysis of the two *RPL3L* variants on each other with regards to atrial fibrillation association in Iceland.

Variant tested	Variant conditioned on	***P*-value**	OR
c.1167+1G>A	p.Ala75Val	7.1x10-4	1.40
p.Ala75Val	c.1167+AG>A	1.6x10-5	1.20

OR = odds ratio.

Supplementary Table 2. Associations of low frequency (<5%) imputed moderate and high impact variants in *RPL3L* with atrial fibrillation in Iceland (N = 14,710) and results of conditional analysis with p.Ala75Val and c.1167+1G>A as covariates.

rsName	marker	Amin	Amaj	Pvalue	OR	Coding effect	Coding change	MAF	Info	Pvalue adjusted	OR adjusted	Covariates
rs140185678	chr16:1953015:S	A	G	6.4×10^{-5}	1.18	missense	p.Ala75Val	3.6	0.99	1	1	p.Ala75Val,c.1167+1G>A
rs140192228	chr16:1945498:S	T	C	8.7×10^{-4}	1.37	splice_donor	c.1167+1G>A	0.6	0.99	1	1	p.Ala75Val,c.1167+1G>A
rs146294352	chr16:1954118:S	T	C	0.0029	1.29	missense	p.Gly12Arg	0.8	0.99	0.0018*	1.32	p.Ala75Val,c.1167+1G>A
rs113956264	chr16:1947003:S	T	C	0.16	1.06	missense	p.Val262Met	4.4	0.99	0.12	1.06	p.Ala75Val,c.1167+1G>A
rs147948209	chr16:1945869:S	C	G	0.20	0.90	missense	p.Ala338Gly	1.1	0.98	0.30	0.92	p.Ala75Val,c.1167+1G>A
rs147972626	chr16:1947063:S	A	G	0.20	1.13	missense	p.Arg242Trp	0.7	0.99	0.13	1.16	p.Ala75Val,c.1167+1G>A
rs146749305	chr16:1954028:S	A	G	0.22	0.02	missense	p.His42Tyr	0.004	0.99	0.23	0.02	p.Ala75Val,c.1167+1G>A
rs75401081	chr16:1947205:S	T	C	0.36	0.02	missense	p.Arg226Gln	0.002	0.95	0.37	0.02	p.Ala75Val,c.1167+1G>A
rs140116056	chr16:1945588:S	T	C	0.42	0.91	missense	p.Val360Met	0.4	0.99	0.41	0.90	p.Ala75Val,c.1167+1G>A
rs141796888	chr16:1947283:S	T	C	0.52	1.05	missense	p.Arg200Gln	1.1	0.98	0.35	1.08	p.Ala75Val,c.1167+1G>A
rs79075024	chr16:1947384:S	G	C	0.59	1.03	splice region	c.502-4G>C	1.8	0.99	0.36	1.06	p.Ala75Val,c.1167+1G>A
rs201602086	chr16:1952995:S	C	G	0.61	0.02	missense	p.Pro82Ala	0.002	1.00	0.62	0.018	p.Ala75Val,c.1167+1G>A
rs201864074	chr16:1954141:S	T	C	0.63	1.97	missense	p.Arg4Gln	0.001	0.96	0.56	2.35	p.Ala75Val,c.1167+1G>A
rs34265469	chr16:1946704:S	A	G	0.73	0.84	missense	p.Pro291Leu	0.03	0.99	0.68	0.81	p.Ala75Val,c.1167+1G>A
rs118144581	chr16:1952959:S	T	C	0.79	0.88	missense	p.Ala94Thr	0.02	0.97	0.86	0.92	p.Ala75Val,c.1167+1G>A

*P-value < 0.0033 (0.05/15). Amin = minor allele, Amaj = major allele, OR = odds ratio, MAF = minor allele frequency.

Supplementary Table 3. Associations of low frequency (<5%) imputed moderate and high impact variants in *MYZAP* with atrial fibrillation in Iceland (N = 14,710) and results of conditional analysis with p.Gln254Pro as covariate.

rsName	marker	Amin	Amaj	Pvalue	OR	Coding effect	Coding change	MAF (%)	Info	Pvalue adjusted	OR adjusted	Covariate
rs147301839	chr15:57632516	C	A	9.03×10^{-6}	1.38	Missense	NP_001018100.1:p.Gln254Pro	1.08	1.00	1.00	1.00	p.Gln254Pro
rs117361082	chr15:57637771	G	A	4.17×10^{-5}	1.31	Missense	NP_001018100.1:p.Glu337Gly	1.38	1.00	0.69	1.06	p.Gln254Pro
rs147173331	chr15:57618043	C	T	0.19	1.15	Missense	NP_001018100.1:p.Leu58Pro	0.54	1	0.15	1.17	p.Gln254Pro
rs146453491	chr15:57633609	T	G	0.25	6.67	Splice region	NM_001018090.6:c.805-4G>T	0.002	0.96	0.24	7.4	p.Gln254Pro
rs142253131	chr15:57604284	A	C	0.31	0.80	Missense	NP_001018100.1:p.Leu31Ile	0.14	0.99	0.36	0.81	p.Gln254Pro
rs138712430	chr15:57618135	A	G	0.56	0.83	Missense	NP_001018100.1:p.Val89Met	0.08	0.98	0.53	0.82	p.Gln254Pro
rs140229874	chr15:57637729	G	A	0.87	0.97	Missense	NP_001018100.1:p.His323Arg	0.2	0.99	0.87	0.97	p.Gln254Pro

Amin = minor allele, Amaj = major allele, OR = odds ratio, MAF = minor allele frequency.

Supplementary Table 4. List of electrocardiogram measures available for analysis of correlation with atrial fibrillation risk variants and sample sizes.

	Sample size: all ECGs included	Sample size: sinus rhythm, excluding atrial fibrillation cases	Number of measurements in each category
Heart rate	88,200	63,000	1
P amplitude	88,100	62,900	12
P area	87,800	62,900	12
P duration	88,000	62,800	12
PR segment	88,200	62,900	12
PR interval	88,100	62,900	12
QRS duration	88,200	63,000	12
R amplitude	88,000	62,900	12
QT interval	88,200	63,000	1
T amplitude	88,100	62,900	12
T area	88,100	62,900	12
T duration	88,100	62,900	12

Supplementary Table 5. Association of three novel atrial fibrillation variants with sick sinus syndrome in 3,568 cases and 346,025 controls from Iceland and 403 cases and 403,181 controls from the UK Biobank.

		***MYZAP* p.Gln254Pro**		***RPL3L* p.Ala75Val**		***RPL3L* c.1167+1G>A**	
	**Number of cases/controls**	**OR (95% CI)**	***P*-value**	**OR (95% CI)**	***P*-value**	**OR (95% CI)**	***P*-value**
**Iceland**	3,568/346,025	1.51 (1.21-1.90)	3.5×10^{-4}	1.01 (0.88-1.17)	0.83	0.97 (0.69- 1.37)	0.85
**UK**	403/403,181	3.80 (1.89-7.62)	1.8×10^{-4}	0.91 (0.62-1.34)	0.62	2.45 (0.93-6.48)	0.070
**Combined**		1.65 (1.33-2.05)	5.0×10^{-6}	1.00 (0.88-1.14)	0.97	1.07 (0.78-1.48)	0.67

OR = odds ratio.

Supplementary Table 6. Association of three novel atrial fibrillation variants in *MYZAP* and *RPL3L* with secondary phenotypes under an additive model.

Phenotype	Number of cases	*MYZAP* p.Gln254Pro missense		*RPL3L* p.Ala75Val missense		*RPL3* c.1167+1G>A splice-donor	
		**OR/*beta**	***P*-value**	**OR/*beta**	***P*-value**	**OR/*beta**	***P*-value**
Sick sinus syndrome	3,568	1.51	0.00035	1.02	0.83	0.97	0.85
Pacemaker implantation	3,578	1.41	0.0031	1.03	0.66	1.31	0.078
Cardioembolic stroke	1,369	1.30	0.17	1.22	0.059	1.46	0.093
Ischemic stroke	5,626	1.09	0.41	1.03	0.60	1.30	0.036
Coronary artery disease	37,782	1.17	0.0096	1.01	0.71	0.997	0.97
Type II diabetes	11,448	0.97	0.73	0.996	0.94	1.01	0.92
Aortic valve stenosis	2,457	1.00	0.98	0.93	0.45	1.28	0.20
Hypertension	54,974	1.00	0.98	0.96	0.22	1.07	0.33
Dilated cardiomyopathy	424	1.28	0.49	0.81	0.34	1.05	0.92
Thyroid stimulating hormone	188,175	-0.01*	0.45	0.002*	0.80	-0.001*	0.95
Ventricular tachycardia	945	1.06	0.81	0.84	0.21	0.67	0.27
Congenital heart disease	2,097	0.88	0.54	1.23	0.071	0.94	0.81
Hypertrophic cardiomyopathy	372	1.28	0.50	0.61	0.074	0.997	1
Heart failure	15,237	1.05	0.49	1.05	0.26	1.26	0.0096
Second and third degree atrio-ventricular-block	1,303	1.26	0.24	0.92	0.50	1.42	0.15

Supplementary Table 7. Association of three novel atrial fibrillation variants in *MYZAP* and *RPL3L* with secondary phenotypes under a recessive model.

Phenotype	Number of cases	*MYZAP* p.Gln254Pro missense		*RPL3L* p.Ala75Val missense		*RPL3* c.1167+1G>A splice-donor	
		OR/*beta	*P*-value	OR/*beta	*P*-value	OR/*beta	*P*-value
Sick sinus syndrome	3,568	4.91	0.060	2.25	0.051	0.017	0.39
Pacemaker implantation	3,578	5.64	0.045	1.05	0.93	0.017	0.37
Cardioembolic stroke	1,369	0.018	0.43	1.34	0.69	0.018	0.61
Ischemic stroke	5,626	0.017	0.13	1.66	0.16	2.06	0.60
Coronary artery disease	37,782	2.52	0.069	1.26	0.34	2.16	0.29
Type II diabetes	11,448	0.38	0.32	0.78	0.53	0.94	0.96
Aortic valve stenosis	2,457	0.018	0.51	0.94	0.93	3.11	0.47
Hypertension	54,974	0.96	0.93	0.81	0.30	1.47	0.56
Dilated cardiomyopathy	424	0.018	0.67	0.018	0.45	0.018	0.73
Thyroid stimulating hormone	188,175	0.067*	0.72	0.048*	0.57	-0.17*	0.47
Ventricular tachycardia	945	5.16	0.20	0.017	0.26	0.018	0.63
Congenital heart disease	2,097	0.018	0.75	1.17	0.86	0.018	0.65
Hypertrophic cardiomyopathy	372	0.018	0.66	0.017	0.32	0.018	0.77
Heart failure	15,237	0.99	0.99	0.92	0.78	1.33	0.78
Second and third degree atrio-ventricular-block	1,303	3.56	0.31	0.017	0.082	0.93	0.99

Supplementary Table 8: Prediction scores for the functional effect of amino acid substitutions and indels from PROVEAN.[2]

	***MYZAP* p.Gln254Pro missense**	***RPL3L* p.Ala75Val missense**
Number of clusters	30	30
Number of supporting sequences used	166	470
PROVEAN score	-4.037	-3.492
Predicted effect	Deleterious	Deleterious
PROVEAN score in RPL3	-	-3.504

Default cutoff for Deleterious classification = -2.5.
Specificity = sensitivity = 80%.

Supplementary Table 9: Characteristics of individuals donating sample from cardiac right atrium for expression analysis.

**Number of samples from right atrium**	167
**Mean age (SD)**	68.8 (12.2) years
**Male**	140/167

Supplementary references

1. Thorolfsdottir, R.B. *et al.* A Missense Variant in PLEC Increases Risk of Atrial Fibrillation. *J Am Coll Cardiol* **70**, 2157-2168 (2017).
2. Choi, Y. & Chan, A.P. PROVEAN web server: a tool to predict the functional effect of amino acid substitutions and indels. *Bioinformatics* **31**, 2745-7 (2015).
