# Supplementary material for: Coding variants in RPL3L and MYZAP increase risk of atrial fibrillation
Source: Commun Biol. 2018 Jun 12;1:68. doi: 10.1038/s42003-018-0068-9 (PMC6123807; doi:10.1038/s42003-018-0068-9)
Supplement: Supplementary file 2 — Description of Additional Supplementary Files [file 42003_2018_68_MOESM2_ESM.docx]

**Description of Additional Supplementary Files**

File Name: Supplementary Data 1

Description: Complete list of associations of *MYZAP* and *RPL3L* variants with electrocardiogram measurements in sinus rhythm of 62,974 individuals, excluding atrial fibrillation cases.
